# Handheld virus concentration method using a hollow fiber filter module

**DOI:** 10.1016/j.mex.2023.102126

**Published:** 2023-03-11

**Authors:** Shinsuke Higuchi, Tatsuki Satou, Yuki Uchida

**Affiliations:** Japan CMC, Vaccine Operations CMC, Global Vaccine Business Unit, Takeda Pharmaceutical Company Limited, 4720 Takeda, Mitsui, Hikari, Yamaguchi 743-8502, Japan

**Keywords:** Syringe based handheld hollow fiber filter module concentration method for virus material, Ultrafiltration, Tangential flow filtration, Concentration, Virus, Hollow fiber filter, Zika virus, Flavivirus

## Abstract

A virus concentration method is required for viral vaccine manufacturing and virus-related research. However, concentration methods, such as ultracentrifugation, often require capital investment. We report a simple and easy-to-use handheld syringe method for virus concentration using a hollow fiber (HF) filter module, which can be applicable to viruses of different sizes, without incorporating any special machines or reagents. This virus concentration method does not use pumps, which might cause shear stress for virus particles; therefore, it is useful for stress-sensitive virus particles, and virus-like particles, as well as other proteins.

The clarified harvest of flavivirus (Zika virus) was concentrated using an HF filter module and compared with a centrifugal ultrafiltration device (CUD) for demonstration of the HF filter method. The HF filter method achieved concentration of the virus solution in less time than the CUD. The yield comparison of the recovered virus solution indicated that recovery from the developed method was comparable to using the CUD, and infectivity was maintained throughout.

• The Zika virus was concentrated from 200 mL to 5 mL within 45 min using the HF filter and handheld syringe module method.
• The handheld HF filter method may be applicable to stress-sensitive viruses and proteins of different sizes.
• The virus concentration process should be conducted in a safety cabinet, which is preferred for virus containment.

## Graphical abstract

**Figure:**
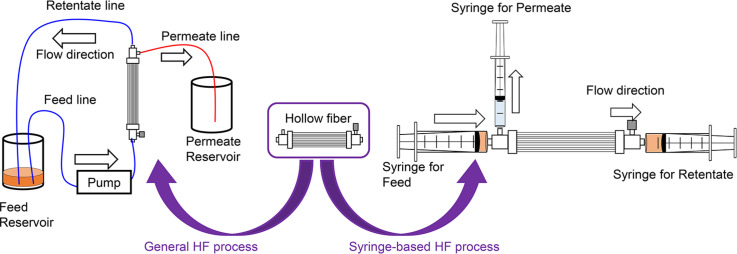

Specifications table

**Table:** 

Subject area:	Immunology and Microbiology
More specific subject area:	Virology
Name of your method:	Syringe based handheld hollow fiber filter module concentration method for virus material
Name and reference of original method:	S. Dasgupta, R. Chavali, N.S.K. Gunda, S.K. Mitra, Hollow fiber concentrator for water quality monitoring: role of surfactant based elution fluids, RSC Adv. 5 (2015)62439-62448. doi: 10.1039/C5RA09662F
Resource availability:	Hollow fiber: https://www.repligen.com/technologies/spectrum-filters “Spectrum MicroKros and MidiKros Hollow Fiber Filters (420-10655-000 Rev5), from Repligen”

## Background

Concentration of virus particles is a key process in both viral vaccine manufacturing and viral characterization research. Commonly used methods for virus concentration are ultracentrifugation, tangential flow filtration, and chromatographic binding and elution [1,2]. Ultracentrifugation has been widely used to concentrate various viruses, although there are many critical parameters that must be optimized before their implementation [3]. With ultracentrifugation, the use of CsCl or sucrose may inactivate sensitive viruses because of their high ionic strength or high osmotic pressure [3]. Moreover, additional steps for removing CsCl, sucrose reagents, or pelleting, and virus resuspension after concentration lead to an increase in unit operations and decrease in yield [1,3]. Furthermore, ultracentrifugation with differential pelleting may cause aggregation, which may be difficult to disaggregate [3]. Finally, ultracentrifugation requires higher investment costs for the ultracentrifuge and rotors compared to those for the ultrafiltration process [4].

Tangential flow filtration using a hollowfiber (HF) filter module is widely used in the vaccine manufacturing field for concentration and buffer exchange because of the low shear stress of virus particles compared with other types of ultrafiltration (UF), such as flat sheet cassettes or tubular membranes [4]. HF UF is generally used in the recirculation mode for virus concentration. To maximize product recovery, the recirculation system is typically flushed with the hold-up volume of rinse buffer after concentrated product collection. Therefore, the hold-up volume, including pumps, tubes, and recirculating bags or bottles, limits the practical final concentration factor of the product to around 50-fold [5].

A previously described HF filter method was developed to detect microbial contamination in water [6]. The authors describe the application of a syringe-based handheld concentration method with a small area of hollow fiber unit (13–41 cm^2^) for detecting *Escherichia coli* in water quality investigations [6]. As shown in the graphical abstract, the syringe-based HF method consists of syringes and a HF filter cassette, which does not use a tube and tube pump recirculation system. This syringe-based method uses syringes for feed flow; therefore, there was no shear stress from the tube pump. In addition, there is no hold-up volume related to the tubing system because the syringes are directly connected to the HF. Therefore, this syringe-based HF filter method can overcome the concerns of shear stress and constraints related to the hold-up volume of the UF recirculation system.

The application of the syringe-based, handheld HF filter method for virus concentration has not been previously described. We have developed and customized a syringe-based HF filter method for virus concentration, which enables a higher concentration factor than previously reported method for water quality monitoring (5-fold for *Escherichia Coli)* [6], and the systems, including filter area and syringes, were scaled up in our method. The original method used surfactants to maximize recovery; however, that approach might inactivate the virus by disrupting its structure [7]. Therefore, virus recovery without surfactants was evaluated using our method to preserve virus structure and infectivity.

To increase the final concentration factor using a syringe-based concentration method, an approximately 10 times larger filter area (115 cm^2^) than the method on which this was based, was used [6]. To our knowledge, this is the first example of a syringe-based concentration method at this scale. In addition, this is the first report of concentrating viruses by manual operation without using any specialist equipment or reagents.

Using this customized method, the hold-up volumes were limited to the HF filter module by directly connecting syringes to both sides of the HF module and performing tangential flow filtration. Therefore, it was possible to minimize the amount of system rinse solution used after the recovery of the concentrated solution. Phosphate-buffered saline (PBS) was used as the final system rinse buffer to minimize any surfactant effects on the virus. For demonstration of the viability of this method, a 40-fold concentration was evaluated using syringe-based HF filter module concentration of a flavivirus (Zika virus). Considering the 50 nm Zika virus size, a 300 kDa nominal molecular weight cut-off (MWCO) HF filter cassette was chosen for this study [8]. Although it was difficult to directly compare the two methods due to the difference of pore size, a 40-fold concentration of the same virus-containing starting material was used for the centrifugal ultrafiltration device (CUD) with a 100 kDa pore sized membrane.

## Methodology for virus concentration using CUD

The methodology and manufacturer for the CUD are described in the supplementary appendix.

## Methodology for virus concentration using HF filter

### Materials

10-layer Nunc EasyFill Cell Factory system (Thermo, 140410)

50 mL Luer lock syringe (Terumo, SS-50LZ)

10 mL Luer lock syringe (NIPRO, 08649)

0.22 μm filter (Sartorius, 5445307H7)

300 kDa hollow fiber (HF) filter, 115 cm^2^ (Repligen, D02-E300-05-S)

100 kDa centrifugal ultrafiltration device (CUD) filter (Millipore Sigma, UFC910024)

1x phosphate-buffered saline (PBS) pH 7.4 (Medicago, 09-9400-100)

Fetal bovine serum (FBS)

Dulbecco's Modified Eagle Medium/Nutrient Mixture F-12 (DMEM/F12)

Water for injection (WFI)

### Procedure

**Fig. 1:**
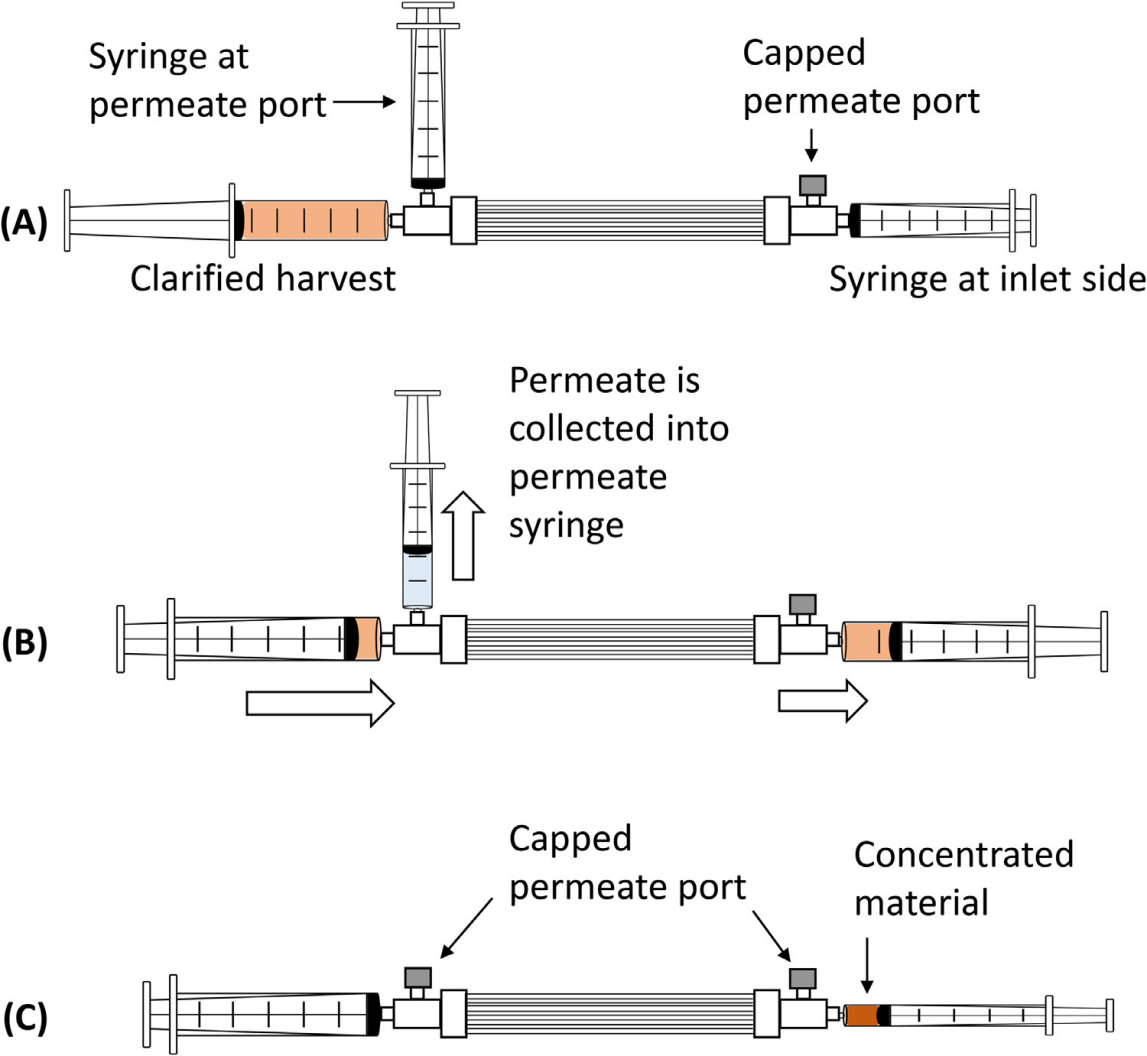
Overview of concentration steps; (A) Clarified virus harvest syringe and other syringes are set up for concentration. (B) Clarified virus harvest is sent to the other side of the syringe, while permeate is collected in the permeate syringe. (C) Concentrated material is collected in a 10 mL syringe with both permeate ports capped.
  3.5. On one side of the inlet syringe clarified virus harvest was injected into the other syringe and the other syringe was moved freely (Fig. 1B). Again, one side of the inlet syringe containing the clarified virus was injected into the other side and this was repeated several times.
  3.6. When the permeate syringe was full, the permeate in the 50 mL syringe was discarded and reconnected.
  3.7. The inlet syringe was re-filled several times with the clarified virus harvest until the entire volume of the prepared clarified virus harvest was filled in the HF module.
  3.8. One of the inlet syringes was changed to a new 10 mL syringe if the concentrated clarified virus harvest volume was less than 50 mL.
  3.9. This was repeated until the clarified concentrate virus harvest volume was 1–2 mL, measured with a 10 mL syringe. The permeate port was then closed with a cap when the concentration was complete.
  3.10. The concentrated clarified virus was harvested in a 10 mL syringe (Fig. 1C).
  3.11. 1x PBS was prepared in a 10 mL syringe for system rinsing and connecting to the HF filter inlet. The required 1x PBS volume was calculated so that the final pool volume was 5 mL.
  3.12. The 1x PBS buffer was injected several times into the other side of the HF module to rinse the HF system.
  3.13. The system rinse was collected into a 10 mL syringe.
  3.14. The concentrated clarified virus harvest and system rinse were pooled.

1. Cell culture and virus propagation
  1.1. Vero cells were cultured in a 10-layer cell factory system until they were confluent in the cell culture medium (DMEM/F12 + 1% FBS).
  1.2. The 10-layer cell factory system was rinsed with a fresh medium (DMEM/F12).
  1.3. Zika virus was inoculated onto the cells, and the virus was propagated for 3 days in the same cell culture medium without FBS (DMEM/F12).
2. Clarification
  2.1. A clarified virus harvest was obtained by filtering the culture supernatant through a 0.22 μm filter.
3. HF module concentration of virus
  As an example, we described the concentration of 200 mL of clarified virus harvest to a final volume of approximately 5 mL.
  3.1. 200 mL of clarified virus harvest was prepared as described above.
  3.2. A new 50 mL syringe was connected at one side of the permeate port. The other side of the permeate port was capped.
  3.3. One of the HF inlets was connected with a new 50 mL syringe.
  3.4. The other inlet was connected to a 50 mL syringe containing the clarified virus harvest (Fig. 1A).

## Method demonstration

200 mL of the clarified virus harvest containing Zika virus was concentrated 40-fold using the HF filter module method (n=3). For comparison, the same volume of clarified virus harvest was concentrated using a CUD (n=1). The HF method took less than 45 min, compared to the CUD which took approximately 1 h.

Clarified virus harvest, concentrated materials, and UF permeates were collected and analyzed for protein concentration, Zika viral RNA copy number, and 50% tissue culture infectious dose (TCID_50_/mL). Zika virus infectivity was evaluated using TCID_50_/mL (Table 1). Results showed TCID_50_ increased from 8.6 log_10_ TCID_50_/mL in the clarified harvest to 9.8 ± 0.2 log_10_ TCID_50_/mL for HF filter method and 9.6 log_10_ TCID_50_/mL fold for the CUD method for the same concentration. TCID_50_ yields were 40 ± 14% for the HF method and 25% for the CUD method, indicating that the HF method was better than the CUD method for concentrating the Zika virus solution, while retaining viral infectivity.

**Table 1:** Log_10_ TCID_50_/mL values of each sample are shown. Samples were serially diluted from 10E^−1^ to 10E^-11^ and inoculated onto confluent Vero cell monolayers in 96-well plates. Each dilution had 8 replicates. After 5 days of incubation, the cytopathic effects of each well were evaluated. Log_10_ TCID_50_/mL was calculated using the Reed and Muench method. For the HF method, mean and standard deviation values were calculated based on experimental triplicates.

Materials	Zika virus titer (log_10_ TCID_50_/mL)
Clarified harvest	8.6
HF concentration (n=3)	9.8 ± 0.2
CUD concentration (n=1)	9.6

Data are presented as mean ± standard deviation

The concentration of Zika viral RNA increased 38.7-fold using the HF method and 31.6-fold using the CUD method (Table 2). The recovery of Zika viral RNA was 95% using the HF method and 79% using the CUD method. Zika viral RNA in the permeate was less than 1.0 x 10^4^ copies/µL for both concentration methods, indicating that Zika virus did not penetrate the 300 kDa HF or 100 kDa CUD membranes.

**Table 2:** The concentration of Zika virus RNA and concentration factors are shown. Zika virus RNA copy number was determined by quantitative real-time PCR. For the HF method, means and standard deviations were calculated based on experimental triplicates. Zika virus RNA was extracted using an RNA isolation kit (Qiagen, 52906), and PCR was performed using a real-time PCR kit (Qiagen, 204443) with Zika virus primer/probe mix (Primerdesign, Path-ZIKV-standard).

Materials	Zika Virus RNA (copies/µL)	Concentration factor
Clarified harvest	3.1 x 10^7^	
HF concentration (n=3)	1.2 x 10^9^ ± 1.1 x 10^8^	38.7 ± 3.5
CUD concentration (n=1)	9.8 x 10^8^	31.6

Data are presented as mean ± standard deviation

In this study, there was no nucleic acid removal process during the preparation of the starting material. Therefore, free viral RNA in the solution and viral RNA contained in the virus particles were counted as Zika viral RNA.

**Fig. 2:**
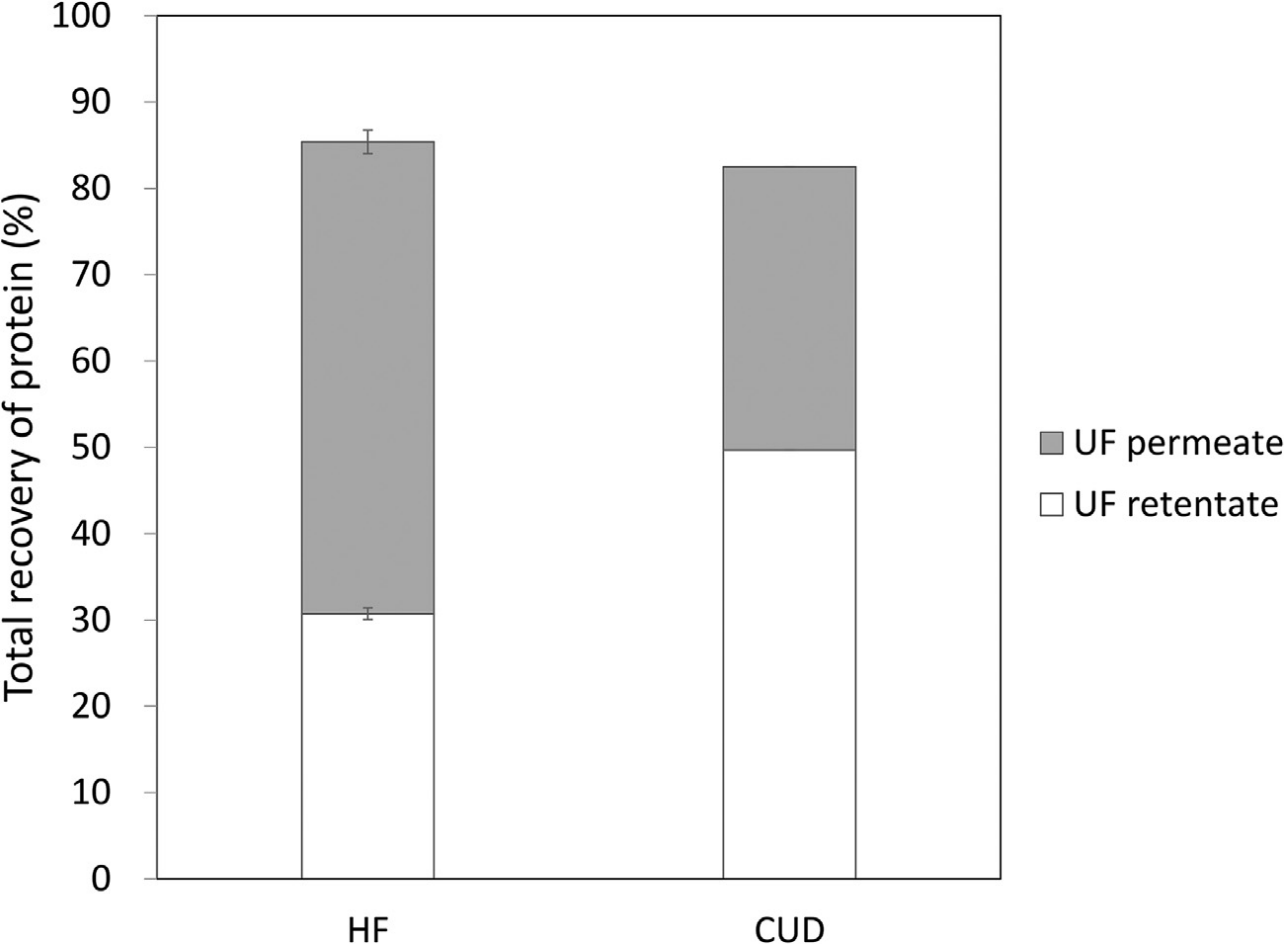
Total protein recovery evaluated using the Bradford assay. The error bar for the HF method shows the standard deviation based on experimental triplicates.

The protein concentration of the HF-processed material, evaluated by the Bradford assay, was lower (UF retentate 1254 µg/mL) than that of the sample processed by the CUD (2028 µg/mL) (Table 3). As the MWCO of the HF filter (300 kDa) was larger than that of the CUD (100 kDa), it is likely that proteins with sizes between 100 and 300 kDa were not retained in the retentate using the HF method, but were retained using the CUD method. The total protein recovery of the UF permeate and UF retentate was more than 80% (Fig. 2), which suggests that protein loss during the concentration procedure was similar between the two methods.

**Table 3:** Total protein concentration was determined by Bradford assay. For the HF method, the mean and standard deviation were calculated based on experimental triplicates.

Sample		Protein concentration (µg/mL)
Clarified harvest		102
HF concentration (n=3)	UF retentate	1254 ± 27
	UF permeate	55 ± 2
CUD concentration (n=1)	UF retentate	2028
	UF permeate	35

Data are presented as mean ± standard deviation

## Conclusion

In this study, a novel handheld HF filter syringe-based concentration method for viruses isolated from clarified harvests was evaluated. To our knowledge, this is the first description of virus concentration without any special reagents or specialist equipment. From the perspective of virus infectivity and viral RNA recovery the HF-based method was confirmed to be more effective than the CUD method. Infectivity data demonstrated that this HF filter method can concentrate viruses with a greater retention of infectious particles than the CUD method. Furthermore, the HF filter method was able to concentrate 200 mL of Zika virus to 5 mL within 45 min without any specialist equipment or reagents, whereas the CUD method took approximately 1 h. Although the maximum MWCO of CUDs is usually 100 kDa, different MWCO filter sizes are available for the HF method. One limitation to our study was that only one test was performed for 100 kDa CUD method compared with triplicate repetition for the 300 kDa HF method, such that further repetitions with same pore sized filter as used in the CUD method would be needed in future studies. Therefore, the handheld HF method is applicable to a wide variety of viruses and proteins. Furthermore, because the virus handling procedures of the HF method are completed only in a safety cabinet, it is useful in terms of virus containment. In conclusion, the handheld HF filter syringe-based method established in this study may be a useful virus concentration tool for use in virus research. However, there is a need for further investigation of the reproducibility for virus concentration, as well as evaluation of different filter sizes, and development of standardization for the HF method.

## CRediT authorship contribution statement

**Shinsuke Higuchi:** Conceptualization, Methodology, Writing – original draft. **Tatsuki Satou:** Writing – review & editing, Supervision. **Yuki Uchida:** Writing – review & editing, Supervision.

## Declaration of Competing Interest

The authors declare the following financial interests/personal relationships which may be considered as potential competing interests:

*Employees of Takeda*

## Data Availability

The data that has been used is confidential.

## Appendix Supplementary materials

Concentration protocol for centrifugal ultrafiltration device Image, application 1
