# Supplementary material for: Handheld virus concentration method using a hollow fiber filter module
Source: MethodsX. 2023 Mar 11;10:102126. doi: 10.1016/j.mex.2023.102126 (PMC10036919; doi:10.1016/j.mex.2023.102126)
Supplement: Supplementary file 1 — Concentration protocol for centrifugal ultrafiltration device [file mmc1.docx]

***Supplementary***

Concentration protocol for centrifugal ultrafiltration device

**Materials and Equipment**

Centrifuge (Kubota, 7780II)

Fixed Angle Rotor (Kubota, AG6512C)

100 kD centrifugal ultrafiltration device (Millipore Sigma, UFC910024)

1xPBS

**Procedure**

- 1. The clarified harvest (100 mL) was subdivided into 10 centrifugal ultrafiltration devices (10 mL each).
  2. The devices were centrifuged at 5000 x *g* for 20 min.
  3. The permeate from the device was collected in a bottle.
  4. An additional 100 mL of the clarified harvest was subdivided into 10 centrifugal ultrafiltration devices (10 mL each).
  5. The devices were centrifuged at 5000 x *g* for 20 min.
  6. The permeate from the device was collected in a permeate bottle.
  7. The residual concentrated material was collected from the devices into one tube with a volume of approximately 4 mL.
  8. The centrifugal ultrafiltration device was rinsed with 1 mL of 1xPBS to a total volume of 5 mL.
  9. The residual concentrated materials and rinses were pooled.
  10. 1xPBS was added to adjust the concentration volume to 5 mL.

Supplier’s protocol link: <https://www.merckmillipore.com/JP/en>

“User Guide, Amicon Ultra-15 Centrifugal Filter Devices for volumes up to 15 mL, PR05035, Rev. 10/18, from Merck.”
